# Radiomics analysis of the optic nerve for detecting dysthyroid optic neuropathy, based on water-fat imaging

**DOI:** 10.1186/s13244-022-01292-7

**Published:** 2022-09-24

**Authors:** Hongyu Wu, Ban Luo, Yali Zhao, Gang Yuan, Qiuxia Wang, Ping Liu, Linhan Zhai, Wenzhi Lv, Jing Zhang

**Affiliations:** 1 Department of Radiology, Tongji Hospital, Tongji Medical College, Huazhong University of Science and Technology, Wuhan, 430030 Hubei China; 2 Department of Ophthalmology, Tongji Hospital, Tongji Medical College, Huazhong University of Science and Technology, Wuhan, 430030 Hubei China; 3 Department of Endocrinology, Tongji Hospital, Tongji Medical College, Huazhong University of Science and Technology, Wuhan, 430030 Hubei China; 4 Department of Medical Imaging, Guangdong Second Provincial General Hospital, Guangzhou, China; 5 Department of Artificial Intelligence, Julei Technology Company, Wuhan, 430030 Hubei China

**Keywords:** Dysthyroid optic neuropathy, Water-fat imaging, Radiomics analysis, Optic nerve

## Abstract

**Objective:**

Detecting dysthyroid optic neuropathy (DON) in the early stages is vital for clinical decision-making. The aim of this study was to determine the feasibility of using an optic-nerve-based radiomics nomogram on water-fat imaging for detecting DON.

**Methods:**

This study included 104 orbits (83 in the training cohort) from 59 DON patients and 131 orbits (80 in the training cohort) from 69 thyroid-associated ophthalmopathy (TAO) without DON patients. Radiomic features were extracted from the optic-nerve T2-weighted water-fat images for each patient. Selected radiomics features were retrained to construct the radiomic signature model and calculate the radiomic score (Rad-score). The conventional MRI evaluation model was constructed based on apical crowding sign, optic-nerve stretching sign and muscle index. The radiomics nomogram model combining the Rad-score and conventional MRI evaluation factors was then developed. Predictive performance of the three models was assessed using ROC curves.

**Results:**

Eight radiomics features from water-fat imaging were selected to build the radiomics signature. The radiomics nomogram (based on Rad-score, apical crowding sign and optic-nerve stretching sign) had superior diagnostic performance than did the conventional MRI evaluation model (AUC in the training set: 0.92 vs 0.80, the validation set:0.88 vs 0.75). Decision curve analysis confirmed the clinical usefulness of the radiomics nomogram.

**Conclusions:**

This optic-nerve-based radiomics nomogram showed better diagnostic performance than conventional MRI evaluation for differentiating DON from TAO without DON. The changes of the optic-nerve itself may deserve more consideration in the clinical decision-making process.

**Supplementary Information:**

The online version contains supplementary material available at 10.1186/s13244-022-01292-7.

**Key points**
1. Radiomics analysis of the optic-nerve on the water-fat imaging has the ability to predict DON patients.
2. Radiomics nomogram of the optic-nerve performed better than conventional MRI evaluation.
3. The features of the optic-nerve may deserve more consideration in predicting DON in the future.

## Introduction

Dysthyroid optic neuropathy (DON) is the most feared complication of thyroid-associated ophthalmopathy (TAO) from optic dysfunction. DON affects approximately 5%-8% of TAO patients [1, 2]. DON can result in irreversible and profound sight loss without a timely diagnosis and intervention [3]. DON is identified, based on its clinical signs and symptoms [3]. In many cases, DON can be insidious and challenging to diagnose in the early stage [1]. Furthermore, TAO patients to present before reaching the peak severity of DON is not common [4]. European Group on Graves’ Orbitopathy (EUGOGO) suggested combining radiological evidence with clinical features to determine a diagnosis of DON [5]. Early diagnosis and treatment are critical for avoiding permanent visual loss. Therefore, the accurate and prompt recognition of DON is crucial for improving the prognosis.

Inflammation, ischemia of the optic nerve and mechanical compression at the orbital apex are the most widely accepted mechanisms of DON [3, 6]. Previous radiological studies have primarily focused on the orbital apex crowding sign (the optic nerve is crowded by the extraocular muscles at the orbital apex) which has been shown to be useful for predicting DON. They ignored the impairment of the optic nerve itself [4, 7–10]. However, several studies have revealed the diameter and the water fraction of the optic nerve are positively correlated with disease activity [11], which further indicates the potential value of the optic nerve in detecting DON patients.

Water-fat imaging can provide uniform and reliable fat suppression and is widely available for clinical application [12–14]. Alexis et al. indicated that water-fat sequence had higher sensitivity, specificity and lower artefacts than did the conventional protocol for assessing TAO [15]. Iterative decomposition of water and fat with echo asymmetric and least-squares estimation (IDEAL) sequence can decompose water and fat into two separate images by using asymmetrically acquired echo and an iterative-squares decomposition algorithm to maximize noise performance [16]. It is suitable for reducing the local static magnetic field inhomogeneity caused by the periorbital and orbital components.

Radiomics can extract and analyze innumerable quantitative image details that cannot be detected with visual inspection by human experts, which has been widely applied in a clinical-decision support system to improve diagnostic accuracy [17, 18]. The prediction of DON by combining the MRI signs of the optic nerve and radiomics analysis may yield a good diagnostic effect. To our knowledge, there is no research investigating the application of radiomics-based model to the differentiation of DON and TAO without DON.

The main objective of our study was to build an optic nerve-based radiomics model on water-fat imaging and to compare its diagnostic performance with that of conventional radiological evaluation for differentiating between DON and TAO without DON.

## Materials and methods

### Study participants

This single-center retrospective study was approved by our Institutional Review Board (IRB ChiECRCT-20170087). A total of 104 orbits from 59 DON patients were included from Tongji Hospital of Huazhong University of Science and Technology (Wuhan, China) between August 2017 and 2020 December. One hundred thirty-one orbits from 69 TAO without DON patients who were treated at Tongji Hospital were also included. They were age-matched with patients with DON. The inclusion and criteria of participants are as follow: (1) age > 18 years, (2) clear refractive media that allowed sufficient image quality, and (3) no treatment with systemic glucocorticoids for at least 3 months before the study. The exclusion criteria of participants are as follow: (1) poor MR image quality; (2) patients with other neurological or ophthalmologic diseases that could explain the vision loss; (3) patients with signs of severe corneal exposure; and (4) patients who had undergone steroid therapy, radiotherapy, or surgical decompression; (5) TAO without DON patients whose age did not match with DON patients.

Thyroid-associated ophthalmopathy (TAO) was diagnosed based on the criteria, established in 1995 by Bartley et al. Dysthyroid optic neuropathy (DON) was made on the basis of the presence of two or more of the following clinical findings: relative afferent pupillary defect when unilaterally affected, color visual defect, reduced visual acuity, papilloedema, visual field defect, and abnormal pattern visual evoked potential test.

Participants were randomly divided into the development and validation sets in an 8:2 ratio. Eighty-three orbits from 44 DON patients and 80 orbits from 40 TAO without DON patients were allocated to the development sets, and the subsequent orbits from participants were allocated to the validation sets. Table 1 shows the detailed demographic information of the participants.

**Table 1:** Demographic and clinical characteristics of the patients in the training and validation cohorts

Characteristics	Training cohort			Validation cohort		
	DON	TAO without DON	*p* value	DON	TAO without DON	*p* value
Age (years)	50.93 ± 8.39	45.9 ± 8.64	< 0.001	52.57 ± 12.17	47.1 ± 9.0	0.039
Patients (n)	46	42		13	27	
Orbits (n)	83	80		21	51	
Sex (M/F)	25/21	19/23	0.522	7/6	18/9	0.439
Euthyroid TAO (n)	5	3		2	2	
Smoker (n)	18	15	0.818	5	9	0.785
Duration of TAO	5.59 ± 3.77	8.93 ± 10.9	< 0.001	6.29 ± 4.33	6.97 ± 4.9	0.58
VA	0.6 (0.5, 1.0)	0.85 (0.8, 1.0)	< 0.001	0.5(0.25, 0.8)	0.8 (0.8, 1.0)	< 0.001
CAS	4 (2, 5)	2 (1, 2.75)	< 0.001	3 (2, 3)	2 (1.5, 2)	0.002
Proptosis (mm)	19 (18, 21)	19 (17.25, 20)	0.058	19 (18, 20)	19 (18, 20)	0.157
MI (%)	62.66 ± 9.04	58.98 ± 10.68	0.019	70.02 ± 9.54	60.67 ± 9.64	< 0.001
Apical crowding sign (n)	56	19	< 0.001	14	13	0.002
Stretching sign (n)	54	17	< 0.001	13	12	0.003

The continuous data are presented as (mean ± standard deviation) or as median (interquartile range)

The* p* values are derived from t-tests, Kruskal tests or chi-square tests

CAS, clinical activity score; DON, dysthyroid optic neuropathy; MI, muscle index; Stretching sign, optic nerve stretching sign; TAO, thyroid-associated ophthalmopathy; VA, visual acuity

### MRI data acquisition

All participants underwent MRI within one week of the clinical diagnosis of DON or TAO. All MRI scans were conducted on a 3.0 T MR machine (Discovery 750; GE Healthcare) using a 32-channel head coil. The patients were placed in the head-first supine position with their eyes closed during the MRI scanning [11]. Coronal IDEAL-T2WI and axial IDEAL-T2WI sequences were obtained, providing water images. The detailed parameters of coronal and axial IDEAL-T2WI sequences are provided in Table 2.

**Table 2:** Axial and coronal IDEAL-T2WI parameters

Parameters	Axial IDEAL-T2WI	Coronal IDEAL-T2WI
TR (ms)	2700	2200
TE (ms)	72	68
Flip angle (°)	111	111
Slice thickness	3	3
Spacing (mm)	0.6	0.6
Matrix size	320 × 224	320 × 224
FOV (mm)	20 × 20	20 × 20
NEX	1	2
TA (min:s)	2:12	2:52
Bandwidth (kHz)	83.33	62.5
Echo train length	18	12

TR: repetition time, TE: echo time, FOV: field of view, NEX: number of excitations, TA: Acquisition time

The conventional MRI assessments were independently conducted by two radiologists (two trained neuroradiologists with more than 5 years’ experience in diagnosing DON). They were blinded to the diagnoses (TAO with DON or TAO without DON). The conventional MRI assessment included:

1. Apical crowding: Crowding of the optic nerve at the orbital apex by enlarged extraocular muscles. It was evaluated on coronal water-IDEAL-T2WI imaging as described by Nugent et al. [19]. (Additional file 1: Fig. S1).
2. Optic nerve stretching: Globe protrusion exceeds the interzygomatic line at least 21 mm. It was evaluated on axial water-IDEAL-T2WI images as described by Rutkowska-Hinc et al. [9].
3. Muscle index (MI): Assessing the ratio of the extraocular muscle meridian to orbital meridian. Using the method described by Lynn Barret et al. [20]. (Additional file 1: Fig. S2)

### VOI delineation and radiomics feature extraction

**Fig. 1:**
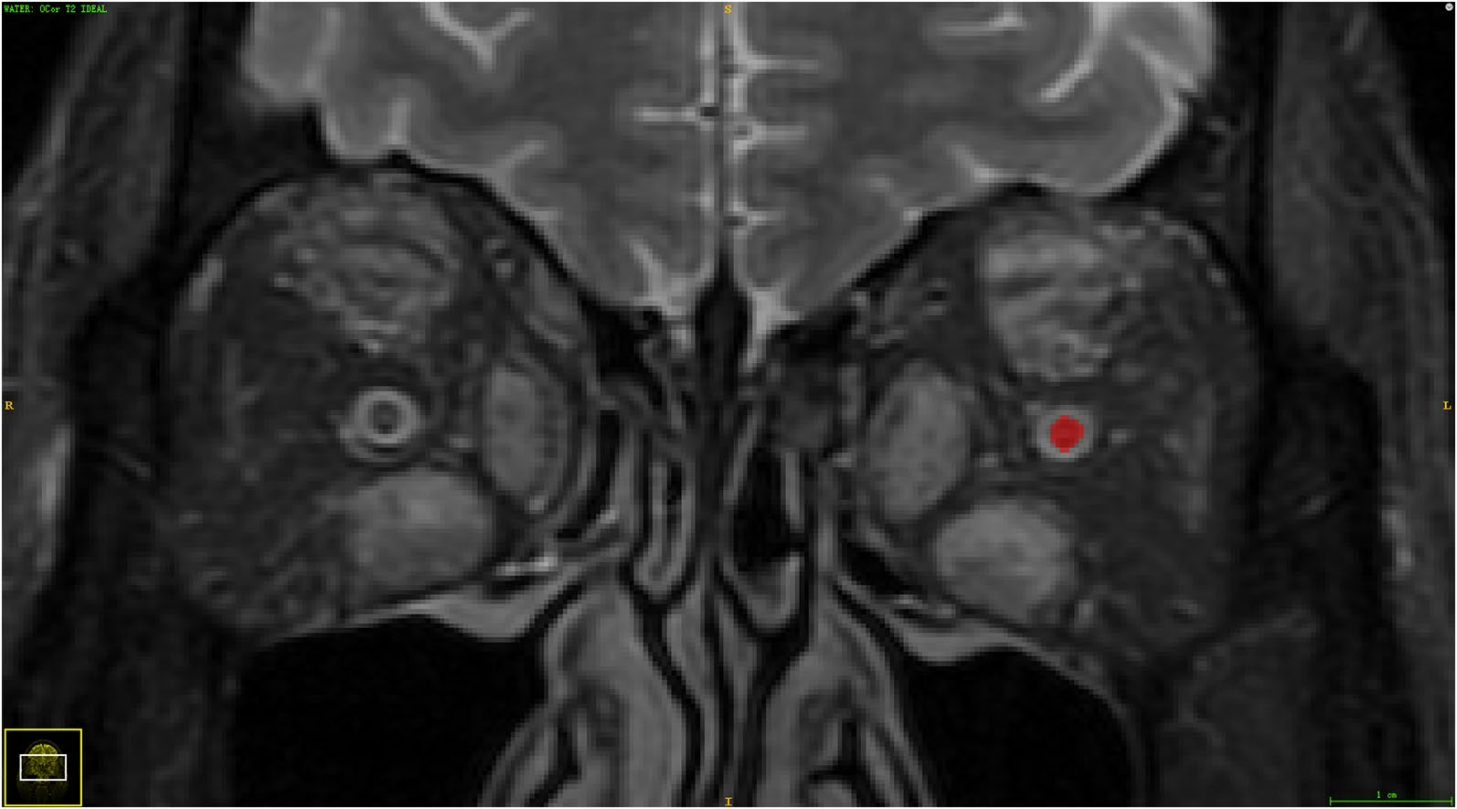
Manual segmentation of the optic nerve tissue in the participants

The VOI (volume of interest) of the optic nerve tissue was manually segmented with ITK-SNAP software (v. 3.6.0; www.itksnap.org) on the coronal water-IDEAL-T2WI images. VOI was drawn on each image slice that contained the optic nerve tissue by two radiologists with more than 5 years of experience in diagnosing DON independently. The radiologists were unaware of the results of diagnoses (TAO with or without DON). The details of radiomics feature extraction are shown in the Supplementary Material (Additional file 1: Appendix E1) (Fig. 1).

### Construction of the radiomic signature

Spearman’s correlation coefficient was applied to calculate the relevance and redundancy of the features. The maximum relevance minimum redundancy (mRMR) algorithm was applied to assess the features. The least absolute shrinkage and selection operator (LASSO) logistic regression with tenfold cross-validation was applied to identify the optimal predictive DON features in the development set. The radiomics score (Rad-score) was built using the selected features, weighted by LASSO logistic regression model.

### Development of radiomics nomogram and the performance of different models

The conventional MRI prediction model was developed using logistic regression. Then, a multivariate logistic regression analysis included the independent conventional MRI factors. The Rad-score was selected to build the radiomics nomogram model. The calibration curve and Hosmer–Lemeshow test were applied to investigate the performance characteristics of the radiomics nomogram. Receiver operating characteristic (ROC) curves were constructed to compare the performances of each model for differentiating DON from TAO without DON. The specificity, sensitivity, accuracy, and area under the curve (AUC) of each model were calculated. The AUCs were compared by using Delong’s test. Decision curve analysis (DCA) was applied to determine the clinical usefulness of the radiomics nomogram and the conventional MRI evaluation model by quantifying the net benefits for different threshold probabilities in the validation set.

### Statistical analysis

Statistical analysis was conducted with R version 3.6.1 and MedCalc version 12.7.0. Interrater and intrarater reliability was assessed by using intraclass correlation coefficient (ICC). *p* < 0.05 were considered as statistical significance level. The packages of R3.6.1 that were used are shown in the Supplementary Material (Additional file 1: Appendix E2).

## Results

### Demographic and clinical data

The participants’ demographic and clinical data in the development and validation cohorts are shown in Table1. There were no significant differences between the two cohorts. For MI measured on IDEAL-T2WI sequence, intrarater agreement was excellent (ICC, 0.97(0.96–0.98)) and interrater agreement was excellent (ICC, 0.93(0.91–0.94)); for apical crowding sign, intrarater agreement was excellent (ICC, 0.94(0.92–0.95)) and interrater agreement was excellent (ICC, 0.90(0.88–0.93)); and intrarater agreement was excellent (ICC, 0.93(0.91–0.94)), interrater agreement was excellent (ICC, 0.91(0.89–0.93)) for optic nerve stretching sign. The MI was significantly higher in DON group than that in TAO without TAO group (*p* = 0.019 in development cohort, *p* < 0.001 in validation cohort). Apical crowding sign occurred in DON patients statistically more often than in TAO without DON patients (*p* < 0.001 in development cohort, *p* = 0.002 in validation cohort). And the optic nerve stretching sign occurred in DON patients statistically more often than in TAO without DON patients (*p* < 0.001 in development cohort, *p* = 0.003 in validation cohort) (Table 1).

### Development of the conventional MRI evaluation model

Significant differences existed between groups with regard to the MI, apical crowding sign and optic nerve stretching sign. Multiple logistic regression analyses showed the apical crowding sign (*p* < 0.05) and the optic nerve stretching sign (*p* < 0.05) remained as independent factors in the conventional MRI model.

### Development of radiomics signature model

**Fig. 2:**
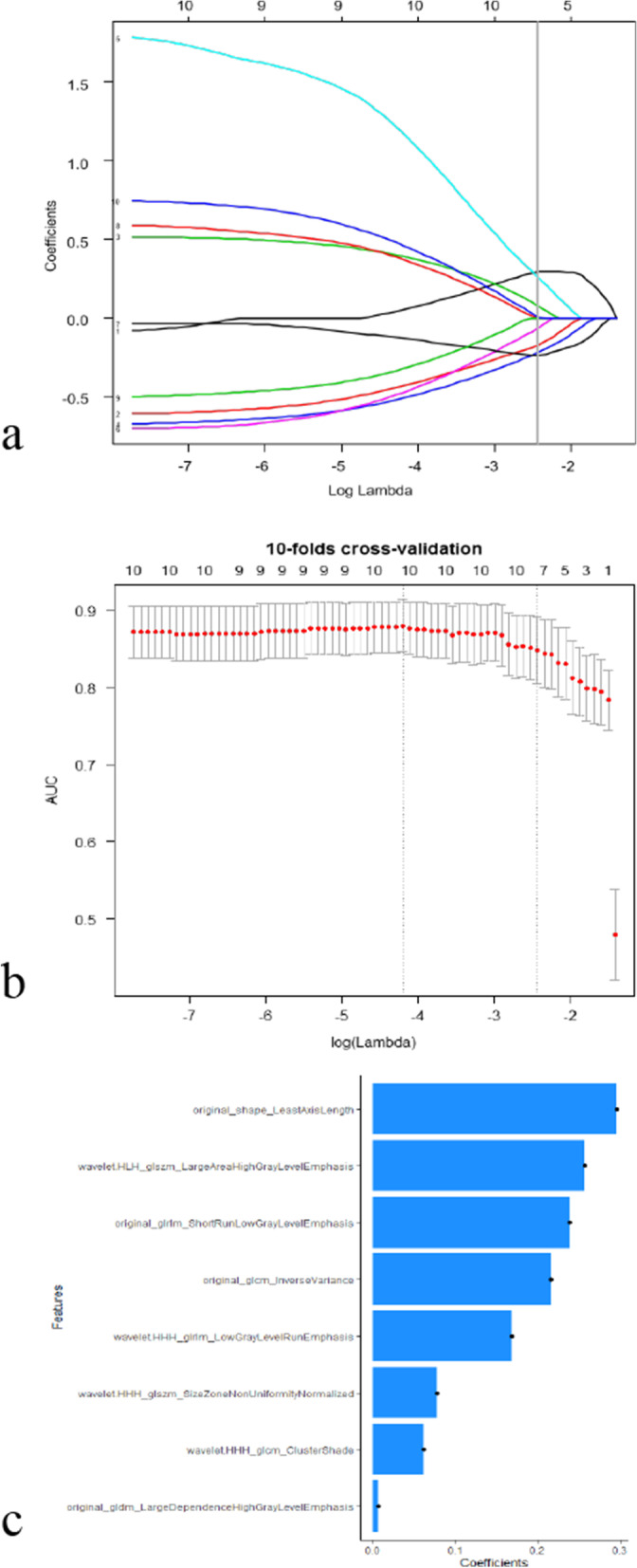
Radiomics features selection using the least absolute shrinkage and selection operator (LASSO) logistic regression. **a**. Tenfold cross-validation of the LASSO logistic regression for the feature selection process. **b**. Eight radiomic features with nonzero coefficients were selected. **c**. Contribution of the selected radiomics features to the model

A total of 851 radiomics features were extracted from IDEAL-T2WI. Eight features with non-zero coefficients after the mRMR algorithm and LASSO algorithm were selected for the radiomics signature model (Fig. 2). The Rad-score calculation was as follows: Rad-score = 0.046222 + (A*0.295835) − (B*0.168698) + (C*0.078249) − (D*0.215994) + (E*0.256508) − (F*0.061405) − (G*0.238402) + (H* 0.006713). The details of the radiomics signature features A to H are shown in Table 3. The performance of Rad-score in inter-observer and intra-observer is provided in Additional file 1: Table S1 and S2, Figure S3 and S4.

**Table 3:** Details of the radiomics signature features

Feature	Name	Intra-ICC	Inter-ICC
A	original_shape_LeastAxisLength	0.975	0.979
B	wavelet.HHH_glrlm_LowGrayLevelRunEmphasis	0.998	0.998
C	wavelet.HHH_glszm_SizeZoneNonUniformityNormalized	0.883	0.879
D	original_glcm_InverseVariance	0.983	0.979
E	wavelet.HLH_glszm_LargeAreaHighGrayLevelEmphasis	0.983	0.985
F	wavelet.HHH_glcm_ClusterShade	0.969	0.976
G	original_glrlm_ShortRunLowGrayLevelEmphasis	0.982	0.981
H	original_gldm_LargeDependenceHighGrayLevelEmphasis	0.886	0.891

Intra-ICC, Inter-observer agreement coefficient; interrater agreement coefficient

### Development of the radiomics nomogram model and the performance of different models

**Fig. 3:**
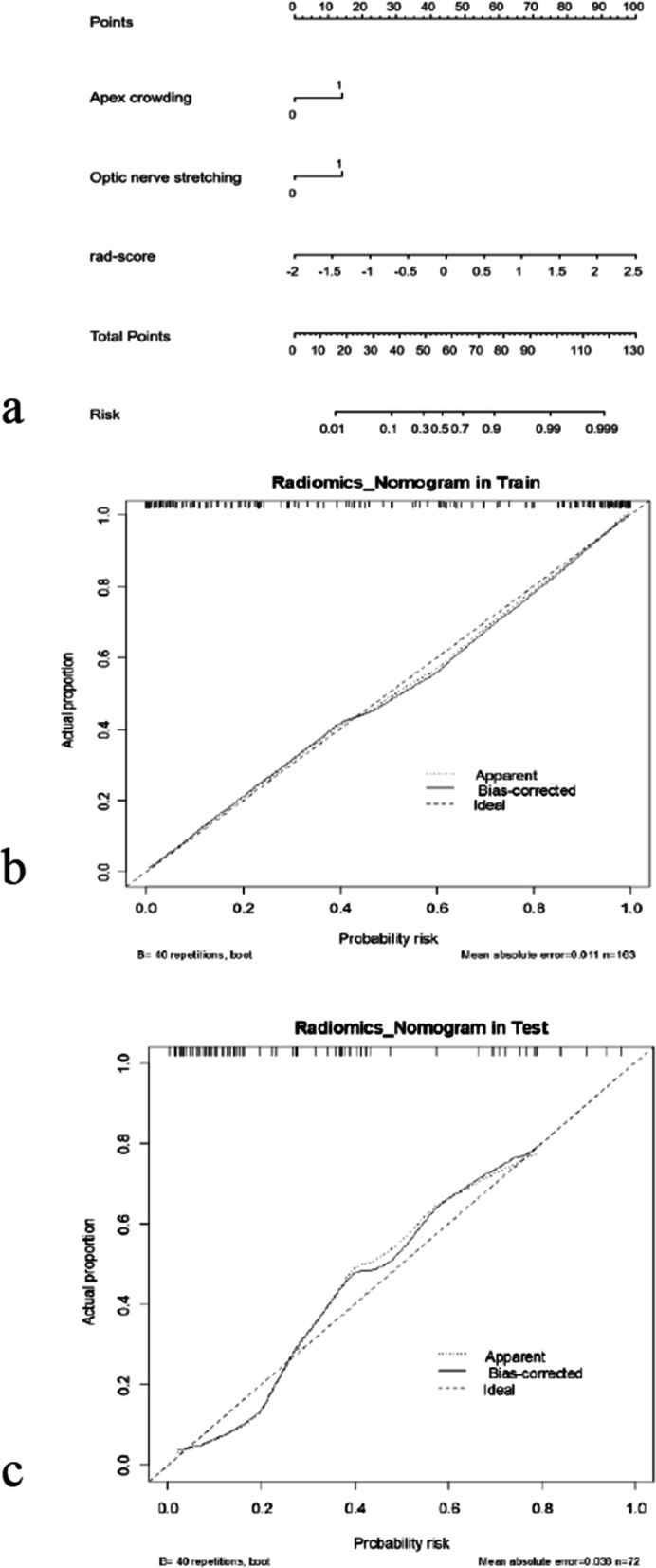
**a**. The radiomics nomogram for the prediction of DON. This was based on the radiomic signature (Rad-score), apex crowding sign and optic nerve stretching sign. **b** and **c** Calibration curves of the radiomics nomogram in the development and validation cohorts. DON, dysthyroid optic neuropathy

The conventional MRI evaluation and Rad-score were integrated to construct a radiomics nomogram model. The radiomics signature, apical crowding sign and the optic nerve stretching sign were independent predictors of DON detection, using a multivariate logistic regression model (Fig. 3a). The calibration curve and Hosmer-Lemeshow test showed good calibration in the training and validation cohorts (Fig. 3b, c). The details of the radiomics nomogram calculation are provided in Supplementary Material (Additional file 1: Appendix E3).

**Fig. 4:**
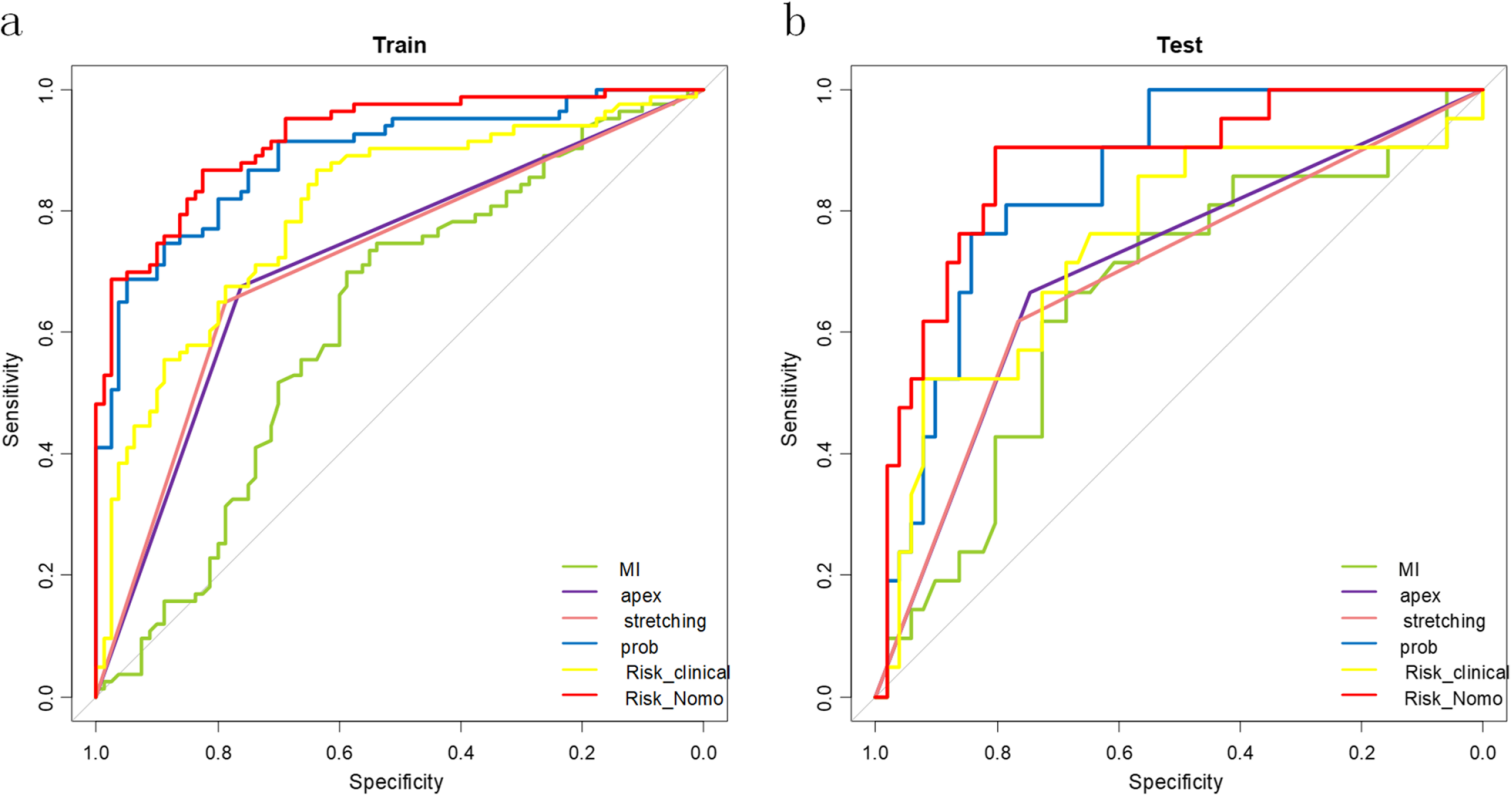
The ROC curves of the radiomics nomogram, radiomic signature (Rad-score), conventional MRI evaluation model, apex crowding sign, optic nerve stretching sign and muscle index (MI) in the development (**a**) and validation (**b**) cohorts. The AUC of the radiomics nomogram in the development and validation cohorts is 0.921 and 0.876, respectively. Apex, apex crowding sign; MI, muscle index; Stretching, optic nerve stretching sign; Convention, conventional MRI evaluation model; Nomogram, radiomics nomogram

Table 4 shows the diagnostic performance of the three models in the validation cohort. The ROCs of the different models are shown in Fig. 4. The AUCs of the three models in the validation set were 0.75 (0.61–0.88) (conventional model), 0.85 (0.76–0.94) (radiomics signature model), 0.88 (0.79–0.97) (radiomics nomogram model), respectively. The sensitivity, specificity, and accuracy of radiomics nomogram were 0.90 (0.76–1.0), 0.71 (0.59–0.82), and 0.76 (0.65–0.86), respectively. The AUC of the radiomics nomogram model was higher than that of the conventional MRI evaluation model (0.88 vs. 0.75, *p* = 0.04, Delong’s test).

**Table 4:** Performance of the conventional MRI evaluation, radiomics signature and radiomics nomogram

Metrics	Training cohort				Validation cohort			
	SEN	SPE	ACC	AUC	SEN	SPE	ACC	AUC
Convention MRI evaluation	0.867 (0.795–0.940)	0.638 (0.525–0.750)	0.755 (0.681–0.819)	0.801 (0.733–0.869)	0.762 (0.571–0.952)	0.608 (0.490–0.745)	0.653 (0.531–0.761)	0.745 (0.608–0.882)
Radiomics signature	0.687 (0.590–0.783)	0.950 (0.900–0.988)	0.816 (0.748–0.872)	0.889 (0.840–0.940)	0.667 (0.476–0.857)	0.843 (0.745–0.941)	0.792 (0.680–0.878)	0.848 (0.758–0.938)
Radiomics nomogram	0.867 (0.795–0.940)	0.825 (0.738–0.900)	0.847 (0.782–0.898)	0.921 (0.881–0.961)	0.905 (0.762–1.000)	0.706 (0.589–0.824)	0.764 (0.649–0.856)	0.876 (0.786–0.966)

SEN, sensitivity; SPE, specificity; ACC, accuracy; AUC, area under the curve

**Fig. 5:**
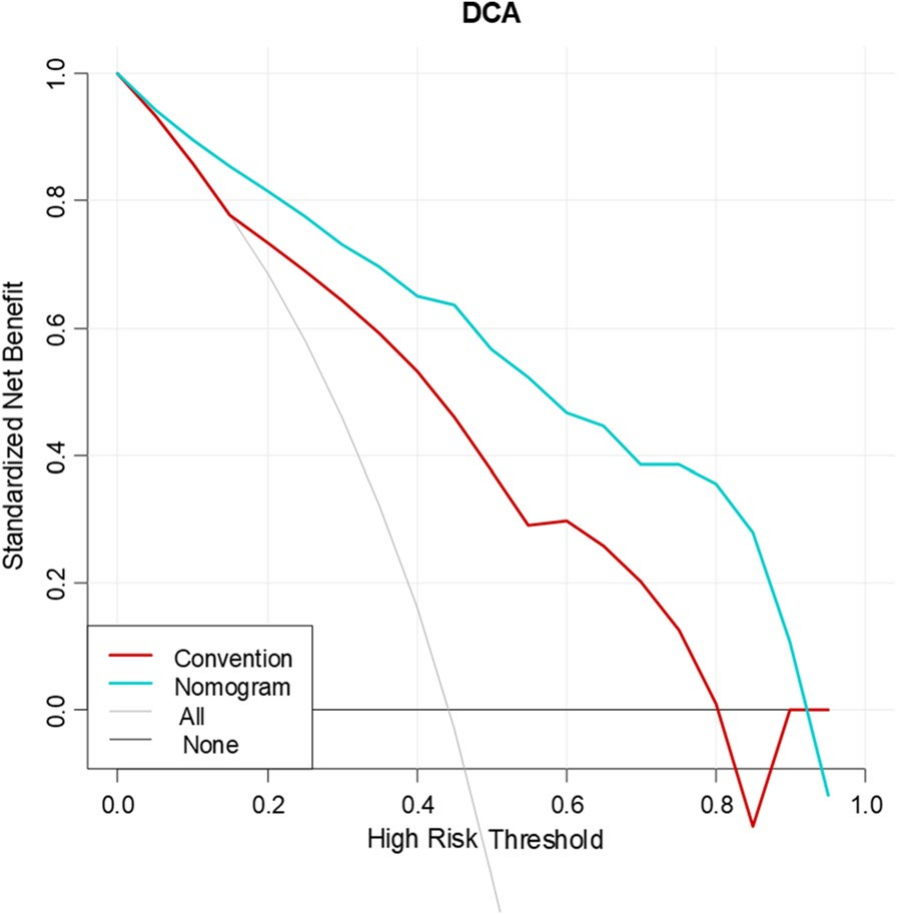
Decision curve analysis (DCA) for the radiomics nomogram model (blue line) and conventional MRI evaluation model (red line). The gray line represents the assumption that all patients are diagnosed with DON. The black line represents the assumption that no patient is diagnosed with DON. The decision curve analysis (DCA) shows that the radiomics nomogram is more advantageous than is the conventional MRI evaluation. Convention, conventional MRI evaluation model; nomogram, radiomics nomogram

The DCA for the radiomics nomogram and conventional MRI evaluation model are shown in Fig. 5. The DCA demonstrated that using proposed radiomics nomogram to predict DON adds more net benefit than does the strategy in which all patients are assumed to have DON or the strategy in which no patients have DON across most of the range of threshold probabilities. This finding indicated the excellent performance of the nomogram model in the clinical decision-making process.

## Discussion

In this study, we developed and validated an optic nerve-based radiomics nomogram on water-fat imaging to provide a noninvasive tool for differentiating DON from TAO without DON. We compared the radiomics nomogram model, radiomics signature model and conventional MRI evaluation model. We summarize our main findings as follows: (1) the radiomics nomogram demonstrated good diagnostic performance in the training and the test cohorts; (2) the radiomics nomogram showed superior predictive performance than using the conventional MRI evaluation model alone; (3) microstructure changes of the optic nerve itself may deserve more consideration for detecting DON, compared to using indirect orbital signs.

We chose the MI, apical crowding sign and optic nerve stretching sign as conventional MRI evaluation methods because the overwhelming majority of imaging studies demonstrated their importance in distinguishing DON from TAO without DON. Based on our results, the conventional MRI evaluation model did not achieve a high AUC (0.75 in the validation set) for differentiating DON from TAO without DON. Moreover, the evaluation of the apical crowding sign and optic nerve stretching sign could be influenced by the reader’s experience. Barrett et al. demonstrated MI < 50% could almost certainly rule out DON. However, MI was not found to significantly contribute to the conventional MRI evaluation model. MI represents a way to quantitatively approximate the orbital apex crowding. The diagnostic performance of MI was not excellent in our study (AUC = 0.62, sensitivity = 0.70, specificity = 0.59, accuracy = 0.644).

To our knowledge, this study is the first application of radiomics analysis based on water-fat imaging in detecting DON. Given the potential value of the optic nerve, we drew the optic nerve tissue on the coronal water-IDEAL-T2WI images. LASSO logistic regression analysis showed that least axis length, large area high gray-level emphasis (LAHGLE) and short run low gray-level emphasis (SRLGLE) were the main three predictive radiomics signature features (The definitions were adapted from http://pyradiomics.readthedocs.io/en/latest/features.html). The least axis length feature yields the smallest axis length of the ROI-enclosing ellipsoid, which is consistent with the orbital apical crowding pathological mechanism in DON. The LAHGLE feature is used to measure the proportion in the image of the joint distribution of large-size zones with higher gray-level values. The SRLGLE feature is used to measure the joint distribution of shorter run lengths with lower gray-level values. Our results revealed the heterogeneity among the gray levels in the optic nerve images between DON and TAO without DON. This finding indicated that microstructural changes of the optic nerve in DON could provide additional information for detecting DON.

In the current study, radiomics nomogram model showed better predictive performance than did the conventional MRI evaluation model, and the Rad-score was retrained as the most important predictive factor in the radiomics nomogram. The DCA indicated the radiomics nomogram was more beneficial compared with the conventional MRI evaluation model. Thus, the radiomics nomogram may assist radiologists in detecting DON and making clinical decisions in a timely manner.

DON is a severe complication of TAO caused by optic nerve dysfunction, and a previous review indicated that no specific features were found in the optic nerve imaging [21]. However, EUGOGO indicated that TAO patients without obvious apical crowding or muscle enlargement also developed DON. Our results showed the optic nerve imaging had good value for the differential diagnosis of DON patients and TAO without DON patients, which suggested that the optic nerve itself may deserve more consideration than the indirect orbital signs in detecting DON in the future.

Our study had several limitations. First, this study was a retrospective single-center study. Second, the sample size was relatively small. The potential selection bias may exist and may have affected the robustness of the prediction model. Prospective multicenter studies with large patient numbers are needed to generate the nomograms from bench to bedside. In addition, our study has only focused on water-fat imaging. Combining the analysis of functional MRI may provide a more comprehensive understanding of the potential value of assessing the optic nerve itself to predict DON.

## Conclusion

We demonstrated the feasibility of developing an optic nerve-based radiomics nomogram for detecting DON in patients with TAO. The radiomics nomogram of the optic nerve could serve as a noninvasive clinical tool in the clinical-decision making process. We believe that the changes of the optic nerve itself deserve more consideration in the task of differentiating DON and TAO without DON in the future.

## Supplementary Information

**Additional file 1**. Additional information of Radiomics feature extraction and Radiomics nomogram calculation formula.

## Data Availability

The datasets used for analyzed during the current study are available from the corresponding author on reasonable request.

## Abbreviations

DCA: Decision curve analysis
DON: Dysthyroid optic neuropathy
IDEAL: Iterative decomposition of water and fat with echo asymmetric and least-squares estimation
LASSO: Least absolute shrinkage and selection operator
MI: Muscle index
TAO: Thyroid-associated ophthalmopathy
