# Supplementary material for: Radiomics analysis of the optic nerve for detecting dysthyroid optic neuropathy, based on water-fat imaging
Source: Insights Imaging. 2022 Sep 24;13:154. doi: 10.1186/s13244-022-01292-7 (PMC9509517; doi:10.1186/s13244-022-01292-7)
Supplement: Supplementary file 1 — Additional file 1. Additional information of Radiomics feature extraction and Radiomics nomogram calculation formula. [file 13244_2022_1292_MOESM1_ESM.pdf]

<u>ELECTRONIC SUPPLEMENTARY MATERIAL</u>

Radiomics analysis of the optic nerve for detecting dysthyroid optic neuropathy, based on water-fat imaging

Appendix E1 Radiomics feature extraction

Radiomics features were extracted by using “PyRadiomics” package (v. 2.2.0) of Python software (v. 3.6.2). The bin width is 25 and resampling is 1.0*1.0*1.0. The radiomics features were extracted from original image and wavelet filter features.

Appendix E2 Packages of R software

The mRMR algorithm was performed using the “mRMRe” package. The LASSO logistic regression, nomograms and calibration curves were performed using the “rms” package. The ROC curves were plotted using the “pROC” package. The DCA was performed using the “rmda” package.

Appendix E3 Radiomics nomogram calculation formula

Radiomics nomogram = -1.351204+ 1.5501629* (apical crowding sign) + 1.5629452* (optic stretching sign) + 2.4893647* (Rad-score)

Table S1. Performance of the Rad-score in different observers in the training cohort.

Metrics	Rad-score				Another observer			
	SEN	SPE	ACC	AUC	SEN	SPE	ACC	AUC
Rad-score model	0.687 (0.578-0.783)	0.950 (0.900-0.987)	0.816 (0.748-0.872)	0.889 (0.839-0.939)	0.663 (0.554-0.759)	0.950 (0.900-0.987)	0.804 (0.734-0.862)	0.873 (0.818-0.929)

Table S2. Performance of the Rad-score in different observers in the test cohort.

Metrics	Rad-score				Another observer			
	SEN	SPE	ACC	AUC	SEN	SPE	ACC	AUC
Rad-score model	0.667 (0.476-0.857)	0.843 (0.745-0.941)	0.792 (0.679-0.878)	0.848 (0.758-0.937)	0.762 (0.571-0.952)	0.803 (0.686-0.902)	0.804 (0.734-0.862)	0.848 (0.749-0.948)

Figure S1. Apical crowding sign of the muscles.

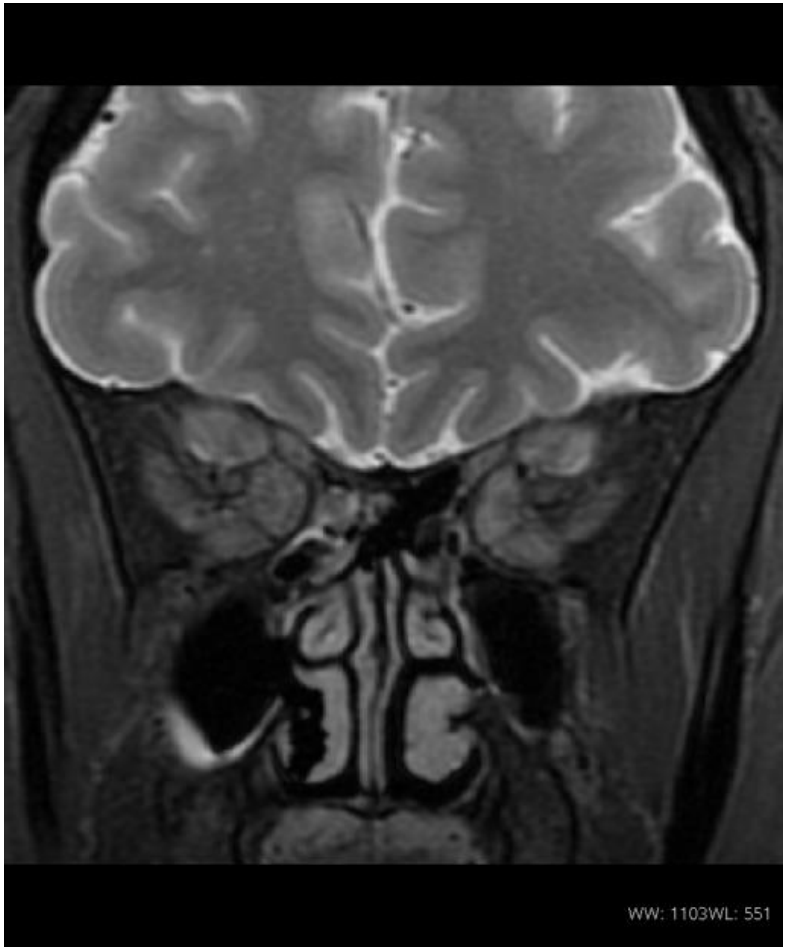

Figure S2. The orbital muscle index (MI) was measured on coronal IDEAL-T2WI images. **a.** The orbital width, meridian of the lateral and meridian of the medial rectus muscles were measured. The horizontal MI = (the meridian of the lateral muscle + the meridian of medial rectus muscle) / the orbital width. **b**. The orbital height, meridian of the inferior rectus muscle and the superior muscle group were measured. Vertical MI = (the meridian of the inferior rectus muscle + the meridian of the superior muscle group) / the orbital height. The largest of the horizontal and vertical MI was considered as the orbital MI.

IDEAL, iterative decomposition of water and fat with echo asymmetric and least- squares estimation; T2WI, T2-weighted.

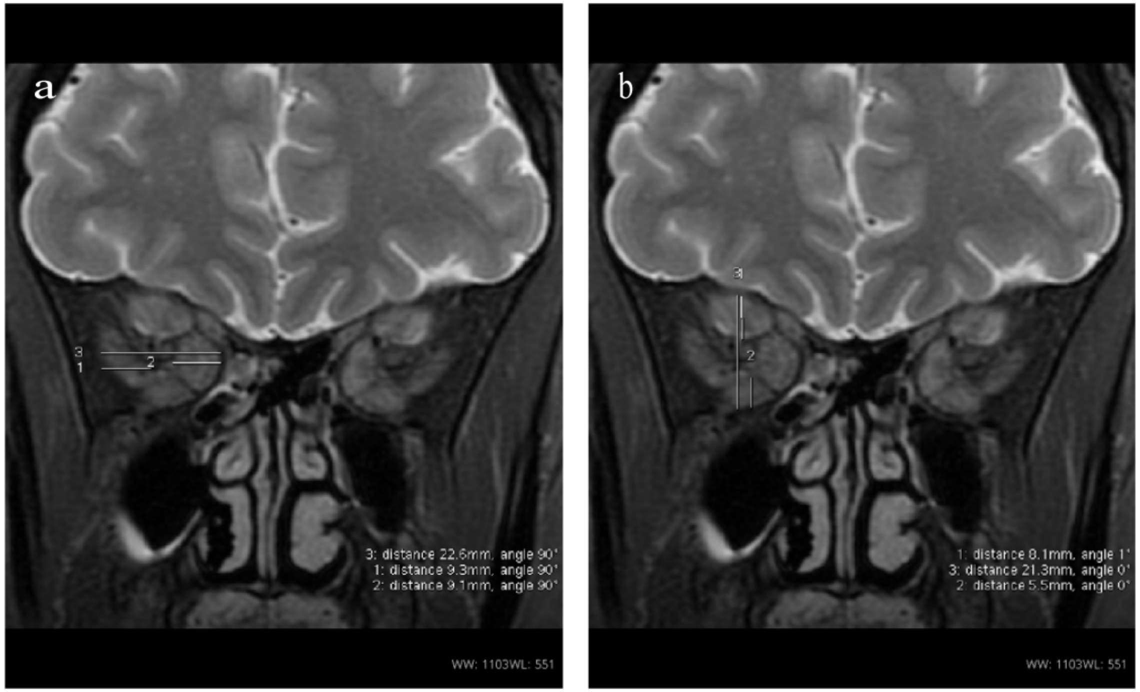
a
3
1
2
3: distance 22.6mm, angle 90°
1: distance 9.3mm, angle 90°
2: distance 9.1mm, angle 90°
WW: 1103WL: 551
b
3
2
1: distance 8.1mm, angle 1°
3: distance 21.3mm, angle 0°
2: distance 5.5mm, angle 0°
WW: 1103WL: 551

Figure S3. Receiver operator characteristic curves of inter-observer and intra-observer in the training cohort.

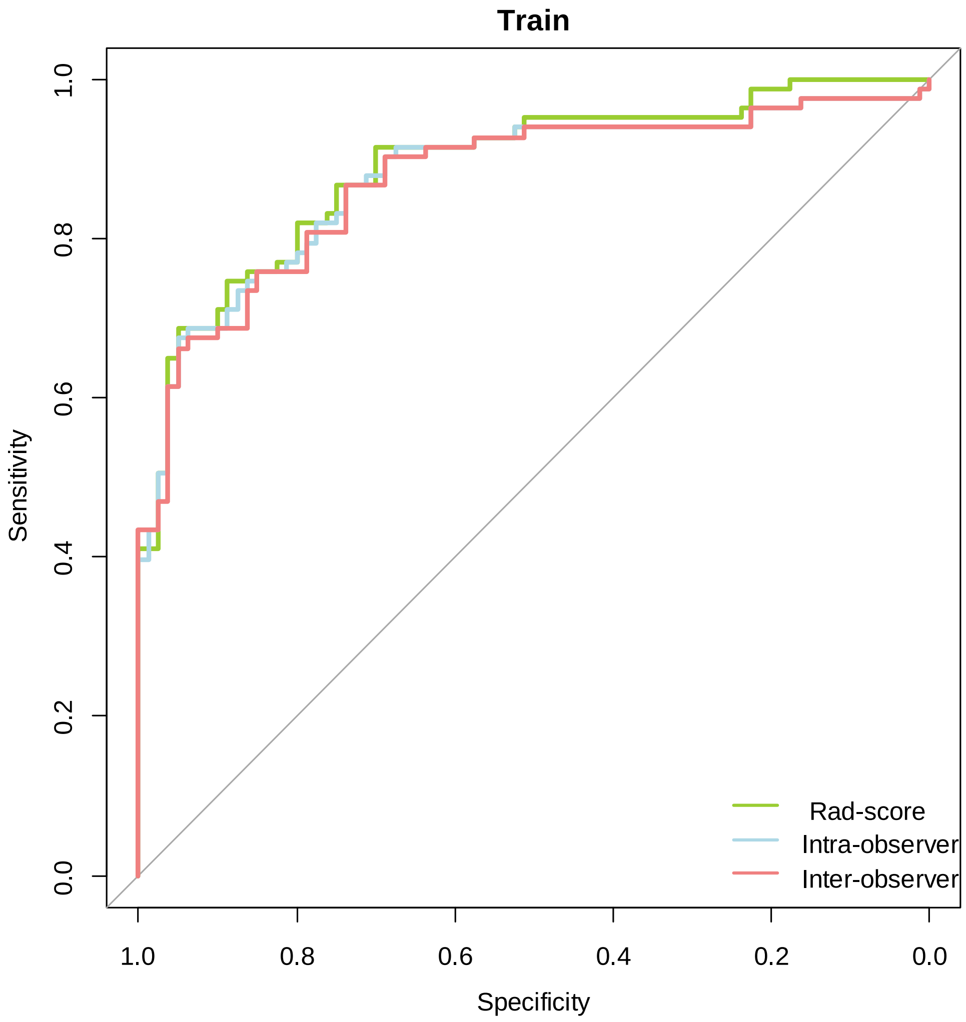

No significant differences between the ROCs of Rad-score, inter-observer and intra-observer were found in the training cohort (Delong's test, $p < 0.05$).

Figure S4. Receiver operator characteristic curves of inter-observer and intra-observer in the training cohort.

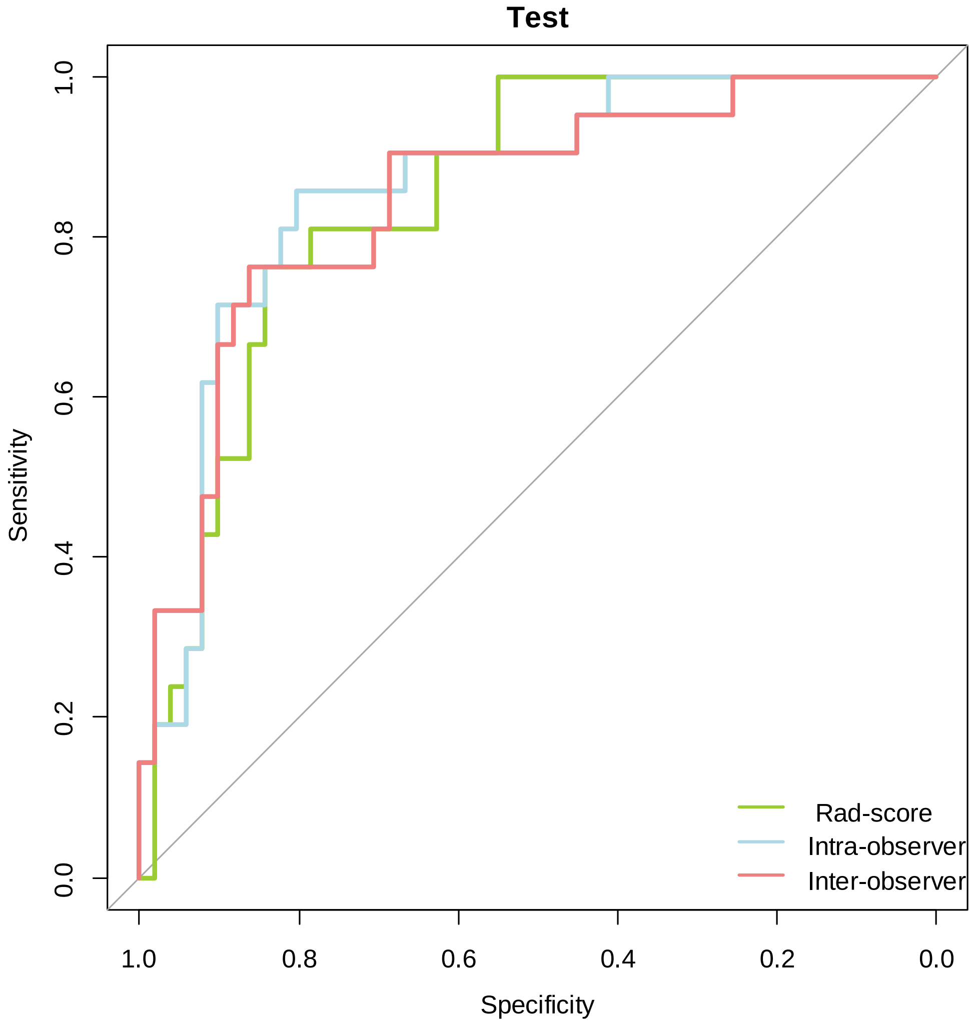

No significant differences between the ROCs of Rad-score, inter-observer and intra-observer were found in the test cohort (Delong's test, $p < 0.05$).
